# Intranasal Dopamine Reduces *In Vivo* [^123^I]FP-CIT Binding to Striatal Dopamine Transporter: Correlation with Behavioral Changes and Evidence for Pavlovian Conditioned Dopamine Response

**DOI:** 10.3389/fnbeh.2016.00080

**Published:** 2016-04-22

**Authors:** Maria A. de Souza Silva, Claudia Mattern, Cvetana Decheva, Joseph P. Huston, Adolfo G. Sadile, Markus Beu, H.-W. Müller, Susanne Nikolaus

**Affiliations:** ^1^Center for Behavioral Neuroscience, Institute of Experimental Psychology, Heinrich-Heine UniversityDüsseldorf, Germany; ^2^M et P Pharma AGEmmetten, Switzerland; ^3^Oceanographic Center, Nova Southeastern UniversityFort Lauderdale, FL, USA; ^4^Department of Experimental Medicine, School of Medicine, II University of NaplesNaples, Italy; ^5^Clinic of Nuclear Medicine, University Hospital DüsseldorfDüsseldorf, Germany

**Keywords:** dopamine, intranasal administration, dopamine transporter, small animal SPECT, Pavlovian conditioning, placebo

## Abstract

**Purpose**: Dopamine (DA), which does not cross the blood-brain barrier, has central and behavioral effects when administered via the nasal route. Neither the mechanisms of central action of intranasal dopamine (IN-DA), nor its mechanisms of diffusion and transport into the brain are well understood. We here examined whether IN-DA application influences dopamine transporter (DAT) binding in the dorsal striatum and assessed the extent of binding in relation to motor and exploratory behaviors. We hypothesized that, based on the finding of increased extracellular DA in the striatum induced by application of IN-DA, binding of [^123^I]FP-CIT to the DAT should be decreased due to competition at the receptor.

**Methods**: Rats were administered 3 mg/kg IN-DA and vehicle (VEH), with IN-DA injection either preceding or following VEH. Then motor and exploratory behaviors (traveled distance, velocity, center time, sitting, rearing, head-shoulder motility, grooming) were assessed for 30 min in an open field prior to administration of [^123^I]FP-CIT. DAT binding after IN-DA and VEH was measured with small animal SPECT 2 h following administration of the radioligand.

**Results**: (1) After IN-DA application, striatal DAT binding was significantly lower as compared to VEH, indicating that the nasally delivered DA had central action and increased DA levels comparable to that found previously with L-DOPA administration; and (2) DAT binding in response to intranasal VEH was lower when IN-DA application preceded VEH treatment. This finding is suggestive of Pavlovian conditioning of DA at the level of the DAT, since the DA treatment modified (decreased) the binding in response to the subsequent VEH treatment. VEH treatment also reduced motor and exploratory behaviors more when applied before, as compared to when it followed IN-DA application, also indicative of behavioral Pavlovian conditioning akin to that found upon application of various psychostimulant drugs.

**Conclusions**: The results: (a) demonstrate a direct central action of intranasally applied DA on the DAT in the dorsal striatum, indicating enhanced DA availability; and (b) provide first evidence of a Pavlovian conditioned DA response at the DAT. The latter results have relevance to understanding neurochemical mechanisms that underlie placebo action in the treatment of Parkinsonian patients.

## Introduction

Deficiencies of dopamine (DA) function play a major role in the pathophysiology of various neurological and psychiatric conditions (for review, see Nikolaus et al., 2007), and also correlate with aging-related deficits in cognitive and emotional processes (for review, see Li and Rieckmann, 2014). DA is known to promote learning and memory (for review, see Myhrer, 2003) and its action has been strongly implicated in the neural mechanisms of information storage and retrieval (Muzzio et al., 2009).

Since DA does not cross the blood brain barrier, its application via the nasal route may be a promising alternative for targeting central nervous system DA receptors (Tayebati et al., 2013). There is evidence that intranasal dopamine (IN-DA) bypasses the blood-brain barrier and thus, can be transported directly from the nasal mucosa along the olfactory pathway into the brain. Application of [3H]-DA into a nostril of mice led to higher DA counts in the ipsilateral olfactory bulb (27 times higher than in the contralateral bulb) and high amounts in the lateral olfactory tract, but not in other brain regions (Dahlin et al., 2000). Similar results were found in rats (Dahlin et al., 2001). Application of IN-DA into the nostrils of anesthetized rats was also shown to increase extracellular DA in the dorsal and ventral striatum for at least 2 h by as much as 250% (de Souza Silva et al., 2008). In the freely moving rat, IN-DA influenced motor activity and exerted antidepressant-like effects (Buddenberg et al., 2008). In aged rats, IN-DA was found to restore deficient object-place memory (Trossbach et al., 2014). In the 6-hydroxydopamine (6-OHDA)-lesion model of Parkinson’s disease (PD), behavioral asymmetries were attenuated by chronic administration of IN-DA (Pum et al., 2009). Moreover, IN-DA reduced behavioral hyperactivity and improved non-selective and selective attention in a rat model of attention-deficit hyperactivity disorder (ADHD), when applied during the prepuberal period (Ruocco et al., 2009, 2014). Intranasal [3H]-DA was also found in the cerebrospinal fluid of monkeys 15 min post-application (Kumar et al., 1976). Neither the route of transportation of IN-DA into the brain, nor its mechanisms of central action are well understood.

We have recently shown that radioligand binding to the striatal dopamine transporter (DAT) is reduced by intraperitoneal (i.p.) application of the DA precursor L-DOPA and that the reduction of DAT binding is related to alterations of motor and exploratory behaviors in the open field (Nikolaus et al., 2013, 2014). In the present study, we jointly assessed neostriatal DAT binding and motor/exploratory behaviors after intranasal application of DA and vehicle (VEH). The rationale was to determine: (a) whether the nasal application of DA influences binding to DAT in the striatum, and thus, to provide information as to its action in the brain; and (b) to assess whether differences in DAT binding are related to differences in various parameters of motor and exploratory activity. For this purpose the animals were tested for 30 min in an open field after application of IN-DA and VEH.

The application of psychostimulant drugs, such as amphetamine and cocaine, is well known to result in Pavlovian-like conditioned behavioral activation and DA sensitization in rodents (Carey and Damianopoulos, 1992; Carey and Gui, 1998; Carey et al., 2005). Even a single drug application can result in long-lasting Pavlovian conditioning (McDougall et al., 2014). In PD patients, conditioned DA release in the striatum of PD patients to placebo treatment was reported using competition for [11C] raclopride binding as measured by positron emission tomography (de la Fuente-Fernández et al., 2001; Lidstone et al., 2010). Therefore, in order to assess the possibility of conditioned changes in DA binding, we employed an experimental design in which each rat was treated with both IN-DA and VEH, five animals with IN-DA preceding VEH, and seven with the order reversed. This procedure permits a comparison of the extent of DAT binding not only between IN-DA and VEH, but also depending on the order in which they were administered. We hypothesized that IN-DA administered prior to VEH would lead to a Pavlovian conditioned increase in DA release to VEH administration and consequently to lower binding of the radioligand to the DAT.

## Materials and Methods

### Animals

We used 12 adult male Wistar rats (TVA, Heinrich-Heine University, Düsseldorf, Germany), weighing 398 ± 58 g (mean ± standard deviation [SD]). Animals were maintained in standard makrolon cages (59 × 38 × 20 cm^3^ animals per cage) in a climate cabinet (Scantainer, Scanbur BK, Karslunde, Denmark; temperature: 20°C; air humidity: 70%) with an artificial light-dark cycle (lights on at 6:00 a.m.; lights off at 6:00 p.m.) and food and water freely available. The study was approved by the regional authority and carried out in accordance with the “Principles of laboratory animal care” (NIH publication No. 86–23, revised 1985) and the German Law on the Protection of Animals.

Each rat underwent 2 trials in randomized order, which were at least 7 days apart. Each trial consisted of: (a) pharmacological challenge with IN-DA or IN-VEH; (b) assessment of behavior in the open-field for 30 min; (c) application of the radioligand 30 min post-challenge; and (d) DAT imaging with small animal SPECT 150 min post-challenge.

### SPECT Camera

The small animal tomograph (“TierSPECT”) was described in detail elsewhere (Schramm et al., 2000). For ^123^I, tomographic resolution and sensitivity amount to 3.4 mm and 16 cps/MBq, respectively. Data were acquired for 60 min in a step-and-shoot mode over a circular orbit in angular steps of 6° (60 projections, 60 s/projection). Data were reconstructed with an iterative ordered-subset-expectation-maximization algorithm (3 iterations, 4 subsets/iteration). No post-filtering procedure was applied. An attenuation correction of 0.11 cm^−1^ was implemented assuming a uniformly attenuating medium.

### DA Treatment

Each rat received treatment with IN-DA and its VEH in randomized trials (IN-DA first in order, *n* = 5; VEH first in order, *n* = 7, with at least 7 days between trials). DA hydrochloride (Sigma-Aldrich, Taufkirchen, Germany) was applied in a dose of 3 mg/kg. The crystalline DA was suspended in a volume of 10 μl of gel composed of a viscous castor oil mixture (M&P Pharma, Emmetten, Switzerland) immediately before usage. It was kept on ice throughout the experiment and protected from light. Per nostril, 5 μl of either DA or VEH was applied with an applicator pipette for viscous liquids (Microman, Gilson, Villiers le Bel, France) and corresponding tips (CP10 Tips, Gilson, Villiers le Bel, France). Drug administration was performed in awake animals over 8 s per nostril with an application depth of 2 mm.

### Behavioral Studies

Immediately after the application of IN-DA or VEH, rats were placed into the center of an apparatus (openfield dimensions: 45 × 45 × 56 cm^3^) with a topunit equipped with light-emitting diodes and a charge-coupled device (CCD) camera (Phenotyper^®^, Noldus Information Technology, Wageningen, Netherlands) situated on the ceiling of the apparatus. Behaviors were assessed with EthoVision XT 8 (Noldus Information Technology, Wageningen, The Netherlands) in blocks of 10 min for a total of 30 min. EthoVision XT 8 automatically determined the distance in cm traveled by the rat and the velocity of locomotion (cm/s) as measures of motor activity as well as the time (s) spent in the center of the open field (a quadratic area of 15 × 15 cm) and the frequency (n) of entries as measures of anxiety. In addition, the durations (s) and frequencies (n) of the following motor and exploratory behaviors were extracted from video recordings by a blinded observer: sitting (total: with or without head-shoulder motility); rearing (freely standing or leaning against the wall) as measure of both motor and exploratory activity; head-shoulder motility (movements of head and shoulders while the animal is sitting) as measure of exploratory activity not associated with locomotion; grooming (licking of fur and paws and scratching). Sitting without any motility was computed from the acquired data (defined as sitting minus head-shoulder motility). The animals were not handled prior to the behavioral studies to adhere to the procedure used in a DAT binding study using L-DOPA (Nikolaus et al., 2013, 2014).

### *In Vivo* Imaging of DAT Binding

Thirty min after IN-DA or VEH application, the animals received i.p. injections of pentobarbital (Narcoren, Merial GmbH, Hallbergmoos, Germany; concentration: 0.5 ml in 1 ml Ringer’s solution; dose: 2 ml/kg). Then 27 ± 4 MBq [^123^I]-FP-CIT (DATSCAN, GE Healthcare, München, Germany; concentration range, 0.07–0.13 μg/ml, specific activity range, 2.5–4.5 × 10^14^ Bq/mmol at reference [REF] time) were administered into the lateral tail vein with a winged infusion needle set (Venofix, B. Braun, Melsungen, Germany and Vacutainer, Beckton, Dickinson & Co, Plymouth, UK). The tube was rinsed with 1 ml 0.9% saline. The total injection volume amounted to 1.3 ml.

Since the equilibrium of [^123^I]FP-CIT binding is reached at 2 h post-injection with the ratio of specific to non-specific striatal uptake remaining stable over the following 2 h (Booij et al., 1997), SPECT measurements were started 2 h after radioligand application. Since SPECT measurements were conducted over 1 h, animals were kept under anesthesia for a total of 3 h.

### Evaluation of DAT Imaging Studies

**Figure 1:**
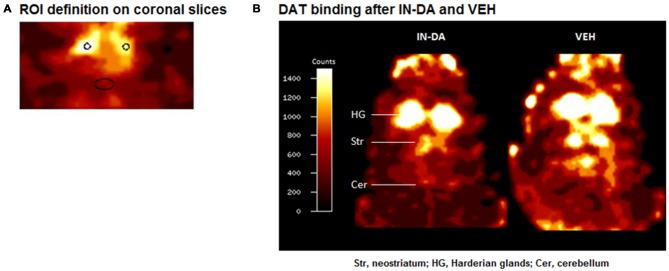
**(A)** ROI definition on coronal slices. **(B)** Coronal [^123^I]FP-CIT images of a rat head after challenge with intranasal dopamine (IN-DA; left) and vehicle (VEH; right). The reduction in striatal dopamine transporter (DAT) binding after IN-DA is clearly visible.

Imaging data were evaluated using the Multi-Purpose-Imaging-Tool (MPI-Tool V3.29, Advanced Tomo Vision GmbH, Kerpen, Germany) as previously described (e.g., Nikolaus et al., 2013, 2014). Briefly, maximum striatal count rates (counts/pixel) were determined on coronal slices by defining a circular region covering an area of 1.5 mm^2^ (Figure 1A). Cerebellar REF count rates (counts/pixel) were obtained by defining an elliptic region (area, 7 mm^2^ comprising 53 pixels) on coronal slices approximately 15 mm posterior to the frontal cortex corresponding anatomically to the rat cerebellum (Figure 1A). Left and right striatal counts rates were averaged. Striatocerebellar ratios were computed as estimates for the binding potential (Laruelle et al., 1994).

### Statistical Analysis

#### DAT Binding

Neostriatal DAT binding as well as motor/exploratory behaviors (traveled distance [cm], velocity [cm/s], duration [s], mean duration [s], and frequency [n] of entries into the center of the open field, sitting with head-shoulder motility, sitting without motility, rearing, head-shoulder motility while sitting, and grooming) were compared between IN-DA and VEH conditions for all animals (*n* = 12) as well as for the animals treated with IN-DA first in order (*n* = 5) and for the animals treated with IN-DA second in order (*n* = 7).

Firstly, DAT binding after IN-DA was compared to DAT binding after VEH (*n* = 12, *t* test for paired samples, two-tailed, *α* = 0.05).

In order to assess the interaction effect of treatment (VEH vs. IN-DA) and order (VEH applied before or after IN-DA) on striatocerebellar DAT binding, two further pairwise comparisons (*t* tests for independent samples, two-tailed, *α* = 0.05) were performed: (1) DAT binding scores after IN-DA of the animals treated with IN-DA first (*n* = 5) were compared to the DAT binding scores after IN-DA of the animals treated with VEH first (*n* = 7); (2) the DAT binding scores after VEH of the animals treated with VEH first (*n* = 7) were compared to the DAT binding scores after VEH of the animals treated with IN-DA first (*n* = 5).

In order to minimize between-subject variance for each comparison (Loftus and Masson, 1994), DAT binding scores in each sample (*n* = 12, *n* = 5, *n* = 7) were adjusted in the following manner: (1) DAT binding scores after IN-DA and VEH were averaged for each sample (*n* = 12, *n* = 5, *n* = 7); (2) DAT binding scores after IN-DA and VEH were averaged for each rat; (3) the mean DAT binding score obtained for each rat was subtracted from the mean DAT binding score obtained for each sample (*n* = 12, *n* = 5, and *n* = 7), thus producing an adjustment factor (i.e., individual deviation from the general level of the dependant variable over both treatments); (4) finally, the adjustment factor was added to each individual DAT binding score in the samples (*n* = 12, *n* = 5, *n* = 7), yielding adjusted samples with equally distributed between-subject differences. From the resulting six samples of adjusted scores (DAT binding scores after IN-DA for *n* = 12, *n* = 5, and *n* = 7; DAT binding scores after VEH for *n* = 12, *n* = 5, and *n* = 7), means and adjusted 95% confidence intervals were computed. The means and adjusted 95% confidence intervals were plotted using SigmaPlot 12.0 (Systat Software GmbH, Erkrath, Germany). The error bars illustrate the pattern of difference between treatment conditions across the samples (*n* = 12, *n* = 5, *n* = 7).

#### Behavior in the Open Field

The interaction effect of treatment and order on behaviors observed in the open field was explored using multivariate simple effects analysis within the framework of the general linear model (3 × 2 within-subject design; within-subject factors: time [0–10 min, 11–20 min, 21–30 min] and treatment [IN-DA vs. VEH]). The behavioral measures included traveled distance, velocity, duration, mean duration and frequency of entries into the center of the open field, as well as duration, mean duration and frequency of the following behaviors: grooming, rearing, head-shoulder motility, sitting with and without head-shoulder motility. The calculations were performed separately for the whole sample (*n* = 12), for the animals treated with IN-DA before VEH (*n* = 5), and for the animals treated with VEH before IN-DA (*n* = 7). The within-subject effect of treatment (IN-DA vs. VEH) and the corresponding F statistic were computed separately for each time bin (0–10 min, 11–20 min, 21–30 min).

Apart from the simple effects analysis, in order to minimize between-subject variance in the error term, an adjustment procedure analog to the one performed on DAT binding scores was executed: (1) for each time bin, behavioral scores after IN-DA and VEH were averaged for each sample (*n* = 12, *n* = 5, *n* = 7); (2) for each time bin, behavioral scores after IN-DA and VEH were averaged for each rat; (3) the mean behavioral score obtained for each rat in each time bin was subtracted from the mean obtained for each sample (*n* = 12, *n* = 5, and *n* = 7) in the respective time bin thus yielding an adjustment factor (one value per subject per time-bin); (4) the adjustment factor was added to each individual score (for each time-bin separately). Finally, for each adjusted sample (per treatment per time-bin: *n* = 12, *n* = 5, and *n* = 7), means and adjusted 95% confidence intervals were computed.

In order to illustrate the pattern of within-subject differences between treatments (IN-DA vs. VEH) as a function of order of application (across the samples: *n* = 12, *n* = 5, and *n* = 7), the means and adjusted 95% confidence intervals were plotted using SigmaPlot 12.0.

#### Correlation Between Neostriatal DAT Binding and Behavior

In order to explore the relationship between the level of DAT binding and the behaviors observed in the open field, we computed two-tailed Spearman’s correlation coefficients (*α* = 0.05) between the striatocerebellar ratio and the behaviors exhibited by the animals in each treatment condition (VEH or IN-DA). Bias corrected and accelerated bootstrap 95% confidence intervals for the correlation coefficients are also reported.

All calculations were performed using IBM SPSS Statistics 22 (IBM Corporation, Armonk, USA).

## Results

### DAT Binding

Figure 1B shows characteristic images of [^123^I]FP-CIT accumulations after treatment with IN-DA and VEH in the same rat. Striatal [^123^I]FP-CIT accumulations were markedly reduced following challenge with IN-DA.

#### REF Count Rates

After IN-DA and VEH, cerebellar radioactivity concentrations (data not shown) amounted to 744 ± 182 counts/pixel (mean ± SD) and 628 ± 217 counts/pixel. No significant differences between conditions were obtained.

#### All Rats Irrespective of Treatment Order (*n* = 12)

After application of IN-DA, maximum striatocerebellar ratios were reduced relative to VEH (paired *t* test: *p* = 0.008).

#### Comparison Between Order of Treatment

Striatocerebellar ratios after IN-DA in animals treated with IN-DA first in order were not significantly different from striatocerebellar ratios after IN-DA in animals treated with VEH first in order.

Striatocerebellar ratios after VEH in animals treated with IN-DA first in order, were lower relative to striatocerebellar ratios after VEH in animals treated with VEH first in order (independent *t* test: *p* = 0.039).

**Figure 2:**
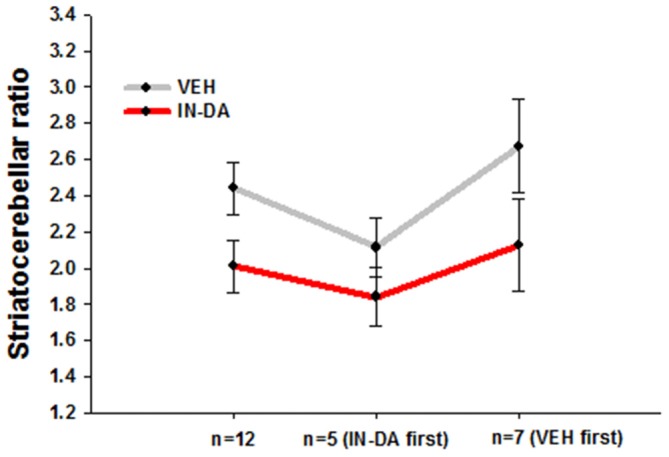
**Striatocerebellar ratios after IN-DA and VEH in all animals (*n* = 12), in animals treated with IN-DA first (*n* = 5) and in animals treated with VEH first (*n* = 7).** Means, adjusted 95% CI of means.

After adjustment of the DAT binding scores in order to reduce between-subject variance within each sample, the resulting means and adjusted 95% confidence intervals amounted to the following values: (1) for all animals (*n* = 12): *M*_VEH_ = 2.44 [95% CI: 2.29, 2.58], *M*_IN−DA_ = 2.01 [95% CI: 1.86, 2.15]; (2) for animals treated with IN-DA first (*n* = 5): *M*_VEH_ = 2.11 [95% CI: 1.95, 2.27], *M*_IN−DA_ = 1.84 [95% CI: 1.68, 2.00]; (3) for animals treated with VEH first (*n* = 7): *M*_VEH_ = 2.67 [95% CI: 2.42, 2.93], *M*_IN−DA_ = 2.13 [95% CI: 1.87, 2.38]. The data are depicted in Figure 2.

#### Differences Between Means

The pattern of within-subject differences in the present study is represented by the distance between the points on the *Y*-axis. The distance in question, representing the effect of the treatment on DAT binding, visibly differs as a function of order: a small difference was obtained from the five animals treated with IN-DA before VEH, while a larger one resulted from the seven animals treated with VEH before IN-DA. When the treatments were compared for all subjects independently of treatment order (*n* = 12), the difference was still present, albeit reduced in relation to *n* = 7 (VEH before IN-DA).

#### Differences Between Adjusted Error Bars

Possible effect of the treatment in future replications of the experiment can be inferred from the error bars. The trend of higher DAT binding after VEH is present independently of the order of treatment. However, the differences between the conditions were smaller in animals treated with IN-DA before VEH. The overlapping error bars for *n* = 5 suggest that it is unlikely to find a significant difference between DAT binding scores when IN-DA treatment precedes VEH. As to the animals treated with VEH first, their difference scores (VEH minus IN-DA) were larger, hence the larger difference between means. However, the trend among them was also more variable, as the larger (less precise) confidence intervals suggest.

### Behavior

#### All Rats Irrespective of Treatment Order (*n* = 12)

The simple effects analysis showed no significant effect of treatment on either behavioral parameter.

#### Rats Treated with IN-DA Before VEH (*n* = 5)

The simple effects analysis revealed no effect of treatment on behavior.

#### Rats Treated with VEH Before IN-DA (*n* = 7)

**Figure 3:**
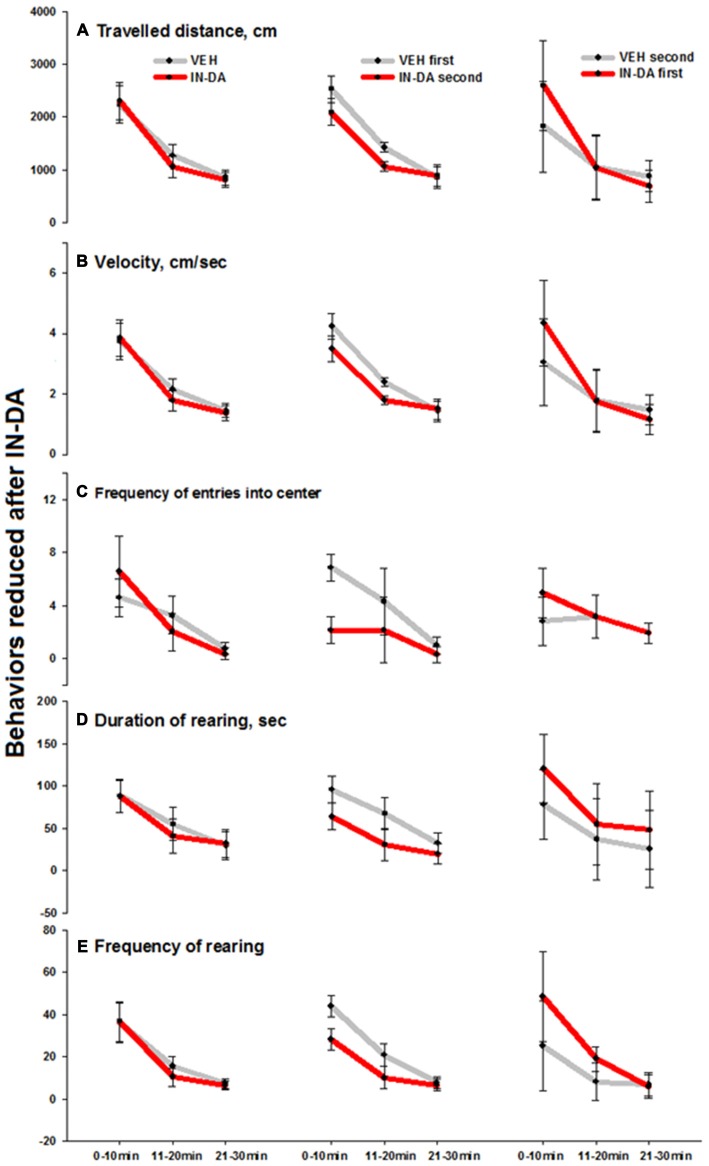
**Behaviors in the open field reduced after IN-DA (vs. VEH as first treatment, *n* = 7). (A)** Traveled distance [cm], 11–20 min. **(B)** Velocity [cm/s], 11–20 min. **(C)** Frequency of entries into center zone [*n*], 0–10 min. **(D)** Duration of rearing [s], 0–10 min. **(E)** Frequency of rearing [n], 0–10 and 11–20 min. Means, adjusted 95% CI of means.

The IN-DA application significantly *decreased* the following behaviors compared to VEH:

- Traveled distance [cm] (*F*_(1,6)_ = 24.4, *p* = 0.003, *M*_VEH_ = 1424.4 [95% CI: 1335.1, 1513.8], *M*_IN−DA_ = 1063.8 [95% CI: 974.5, 1153.1]) and velocity [cm/s] *F*_(1,6)_ = 26.9, *p* = 0.002, *M*_VEH_ = 2.4 [95% CI: 2.3, 2.6] *in time bin 11–20 min*.
- Frequency of entries into the center of the open field [n] (*F*_(1,6)_ = 34, *p* = 0.001, *M*_VEH_ = 6.9 [95% CI: 5.9, 7.9], *M*_IN−DA_ = 2.1 [95% CI: 1.2, 3.1]) *in time bin 0–10 min*.
- Duration of rearing [s] (*F*_(1,6)_ = 6.2, *p* = 0.047, *M*_VEH_ = 95.7 [95% CI: 80.2, 111.3], *M*_IN−DA_ = 64 [95% CI: 48.5,79.6]) *in time bin 0–10 min*.
- Frequency of rearing [n] *in time bins* 0–10 min (*F*_(1,6)_ = 14.1, *p* = 0.01, *M*_VEH_ = 44, [95% CI: 38.9,49.1], *M*_IN−DA_ = 28.3; [95% CI: 23.2, 33.4]) *and 11–20 min* (*F*_(1,6)_ = 6.2, *p* = 0.047, *M*_VEH_ = 20.9 [95% CI: 15.5,26.2], *M*_IN−DA_ = 10 [95% CI: 4.7, 15.3]). The effect was less pronounced on the duration of rearing (0–10 min) and frequency of rearing (11–20 min). Means and adjusted 95% confidence intervals are depicted in Figures 3A–E (traveled distance: Figure 3A, velocity: Figure 3B, frequency of entries into center: Figure 3C, duration of rearing: Figure 3D, frequency of rearing: Figure 3E).

**Figure 4:**
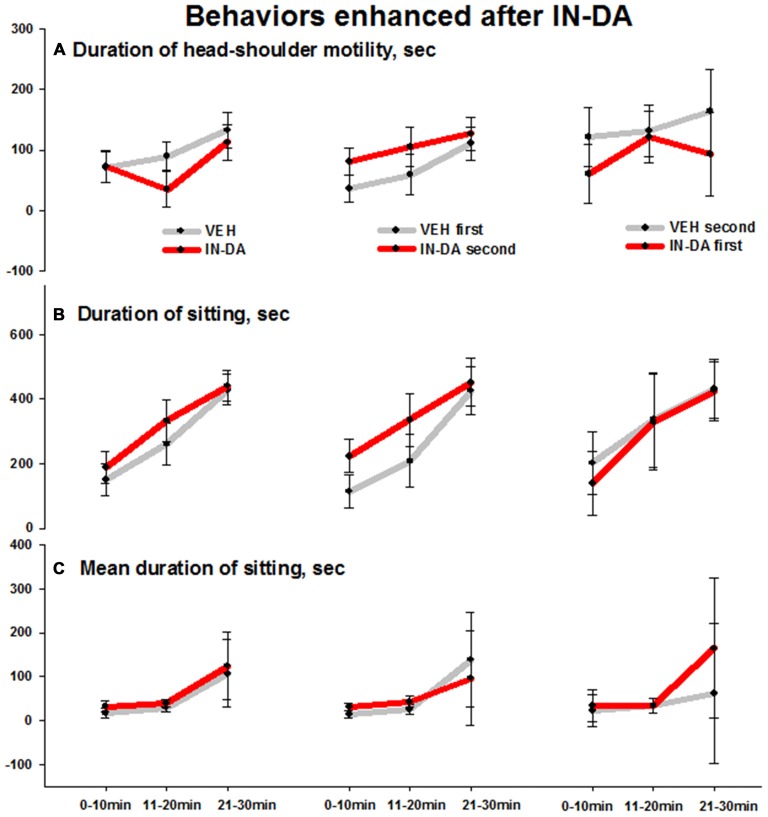
**Behaviors in the open field enhanced after IN-DA (vs. VEH as first treatment, *n* = 7). (A)** Duration of head-shoulder motility [s], 0–10 min. **(B)** Duration of sitting [s], 0–10 min. **(C)** Mean duration of sitting [s], 0–10 min. Means, adjusted 95% CI of means.

IN-DA application significantly increased the following behaviors in comparison to VEH: duration of head-shoulder motility [s] (*F*_(1,6)_ = 6.3, *p* = 0.046, *M*_VEH_ = 35.9 [95% CI: 14, 57.9], *M*_IN−DA_ = 80.8 [95% CI: 58.9, 102.8]), duration (*F*_(1,6)_ = 6.7, *p* = 0.042, *M*_VEH_ = 113.5 [95% CI: 61.2, 165.7], *M*_IN−DA_ = 223.8 [95% CI: 171.6, 276.1]) and mean duration (*F*_(1,6)_ = 7.6, *p* = 0.033, *M*_VEH_ = 14.2 [95% CI: 6.3, 22], *M*_IN−DA_ = 31.9 [95% CI: 24.1, 39.8]) of sitting [total: with or without head-shoulder motility, s] *in time bin 0–10 min*. The effects (illustrated by the error bars) were relatively small. Means and adjusted 95% confidence intervals are shown in Figures 4A–C (duration of head-shoulder motility: Figure 4A, duration of sitting: Figure 4B, mean duration of sitting: Figure 4C).

The fact that IN-DA yielded significant results only when IN-DA followed VEH, can be interpreted in terms of the variability of difference scores, i.e., only when the VEH treatment was not preceded by IN-DA, the trend of the difference was clear enough to produce a significant result. The largest effects were found in four behaviors, which were *reduced after IN-DA treatment*: traveled distance *in time bin 11–20 min* (reduced by 25%, or 361 cm, relative to *M*_VEH_), velocity *in time bin 11–20 min* (reduced by 25%, or 0.6 cm/s, relative to *M*_VEH_), frequency of entries into the center zone *in time bin 0–10 min* (reduced by 69%, or 4.7 entries, relative to *M*_VEH_), and frequency of rearing *in time bin 0–10 min* (reduced by 36%, or 15.7 rearing sequences, relative to *M*_VEH_).

### Correlations Between Neostriatal DAT Binding and Behaviors

#### Correlations After IN-DA Treatment Independent of Order of Treatment (*n* = 12)

**Figure 5:**
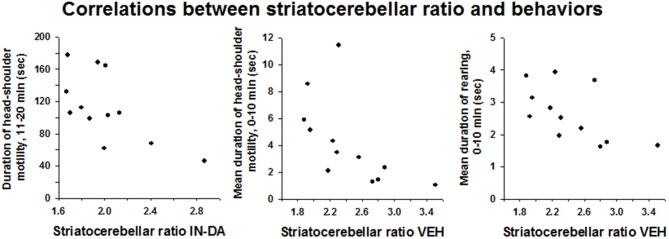
**Correlations between striatocerebellar ratio and behaviors observed after IN-DA or VEH.** Duration of head-shoulder motility [s], 11–20 min, mean duration of head-shoulder motility [s], 0–10 min and mean duration of rearing [s], 0–10 min.

There was a significant inverse relationship between the striatocerebellar ratio and the duration of head-shoulder motility, min 11–20, after IN-DA treatment: *r*_s_ = −0.587, 95% BCa CI [−0.913, −0.011], *p* = 0.045 (Figure 5). Apparently, a higher release of DA (leading to a higher reduction of DAT binding) after IN-DA, predicts a longer duration of head-shoulder motility.

#### Correlations After VEH Treatment Independent of Order of Treatment (*n* = 12)

After VEH treatment, the striatocerebellar ratio correlated negatively with the mean duration of head-shoulder motility from min 0–10 (*r*_s_ = −0.72, BCa CI [−0.978, −0.208], *p* = 0.008) and with the mean duration of rearing, min 0–10 (*r*_s_ = −0.699, BCa CI [−0.935, −0.213], *p* = 0.011). Thus, presumably, a higher release of DA (leading to a higher reduction of DAT binding) after VEH treatment predicts a longer duration of both head-shoulder motility and rearing behavior.

## Discussion

The major results of this work are: (1) that intranasally applied DA reduced DAT binding in the neostriatum, thus demonstrating a central action and indicating enhanced DA availability; and (2) DAT binding upon intranasal vehicle application was higher when preceded by intranasal DA treatment, suggestive of Pavlovian conditioning of the DA response at the DA transporter.

### Neostriatal DAT Binding

Challenge with 3 mg/kg IN-DA significantly reduced neostriatal DAT binding relative to IN-VEH. Cerebellar radioactivity concentrations did not differ between IN-DA and VEH, indicating that no confounding effects were exerted on radioligand accumulation, for instance, by affecting cerebral perfusion.

Neostriatal DAT binding can be altered by treatment with compounds that increase synaptic DA levels in healthy animals as well as in Parkinsonian patients and in animal models of PD with subtotal DAergic depletion (for reviews, see Winogrodzka et al., 2005; Nikolaus et al., 2016). The present effects of IN-DA are consistent with previous *in vivo* and *ex vivo* findings obtained in rats after treatment with L-DOPA (Gnanalingham and Robertson, 1994; Dresel et al., 1998; Sossi et al., 2010; Nikolaus et al., 2013, 2014). L-DOPA is a DA precursor and increases the efflux of DA (for review, see Misu et al., 1996); therefore, the reduction of DAT binding can be interpreted in terms of an increase in levels of DA in the synaptic cleft. Application of DA into the nostrils of anesthetized rats has previously been shown to increase neostriatal DA levels for at least 2 h by as much as 250% (de Souza Silva et al., 2008). These findings suggest that the striatal DA transporter is targeted by either a direct local action of the intranasally applied DA in the synaptic cleft, or by an indirect action involving the stimulation of DA synthesis or release, or even by remote effects elsewhere. The possibility of up-regulation of DAT binding sites at the presynaptic terminals in response to an increase in DA release is unlikely, since IN-DA decreased DAT binding. Moreover, DA levels are likely to have exceeded the capacity of available binding sites at the time [^123^I]FP-CIT was administered.

The K_i_ value of [^123^I]FP-CIT amounts to 3.5 nM under competition with GBR12909 (Neumeyer et al., 1996). For DA, K_i_ values between 0.59 nM and 19 nM were reported using various WIN 35,065 analogs as competitors (Kotian et al., 1996). However, also K_i_ values in the micromolar range were obtained when unlabeled DA was used as a competitor (e.g., Lee et al., 1996). This allows for a tentative estimation of binding relations at the presynaptic terminal. If the affinities of exogenous and endogenous ligands lie within the same order of magnitude, the observed 17% decrease of [^123^I]FP-CIT binding should reflect a just as high increase of synaptic DA. Interestingly, the *in vivo* microdialysis findings of de Souza Silva et al. (2008) showed a 250% increase of endogenous DA at about 30 min after IN-DA administration. This result suggests that the affinity of [^123^I]FP-CIT for the DAT is at least one order of magnitude higher than the affinity of DA with a 17% decrease of [^123^I]FP-CIT binding, reflecting an increase of synaptic DA by about 10- to 15-fold.

The mechanisms by which intranasal DA is transported to the brain from the nasal mucosa are barely understood. Unilaterally applied intransal [3H]DA has been shown to be carried to the ipsilateral olfactory bulb in rodents, likely via the olfactory pathway (Dahlin et al., 2000, 2001). However, various mechanisms of transport from the nose to the brain, including the trigeminal nerves have been proposed (for review, see Tayebati et al., 2013). IN-DA applied in the same dose as in our present study was found to increase the release of DA in both, the nucleus accumbens and striatum of rats, implying both, the mesocorticolimbic and nigrostriatal DA systems (de Souza Silva et al., 2008). The present finding of a decrease in DAT binding in the dorsal striatum after IN-DA treatment significantly adds to our understanding of the central effects of IN-DA, although it has not been determined which DA receptors are responsible for its behavioral actions in the brain, nor whether pre- and postsynaptic receptors are directly or indirectly activated by the nasally applied DA.

### Pavlovian Conditioning of Neural DA Response?

DAT binding in response to VEH administration differed depending on whether VEH preceded followed the application of IN-DA. Striatal DAT binding was significantly lower (and DA levels presumably higher) when IN-DA precede VEH, than* vice versa*. This result can be interpreted in terms of Pavlovian drug conditioning, whereby in the open field, the handling and nasal injection procedure acquired conditioned stimulus properties that led to enhanced release of DA subsequent to the unconditioned DA action of IN-DA and the resulting decrease of exogenous radioligand binding to the striatal DAT. Comparable evidence for conditioned psychostimulant effects at the level of the DAT has not been reported before. Although these differential binding effects dependent on order of presentation could potentially have other causes besides classical conditioning of the drug effects (see below) and require confirmation with adequate control experiments, the possibility of such an action justifies our tentative interpretation.

It should be noted that IN-DA, like most psychostimulants, is likely to have affective, i.e., rewarding properties, although IN-DA has not yet been tested for its potentially rewarding effects. It is well known that cues associated with rewards, including drugs of abuse, such as cocaine, opioids and alcohol can acquire motivational properties and serve as incentives (Robinson et al., 2014). During Pavlovian conditioning of a tone CS with food UCS, DA release in the ventral striatum increased during acquisition of conditioning and decreased during extinction (Sunsay and Rebec, 2014; Biesdorf et al., 2015). Similarly, Ostlund et al. (2014) found increased DA release in the nucleus accumbens triggered by the presentation of a cue that had been paired with cocaine reward. In fact, the mechanisms that underlie Pavlovian drug conditioning in general are likely to involve dopaminergic mechanisms (Sparks et al., 2014; Lopez et al., 2015), and the nucleus accumbens and dorsal striatum is thought to play a focal role in such conditioning (Darvas et al., 2014). Reward-predictive cues were found to evoke responses of neurons in the NAc (Ambroggi et al., 2011) and changes in striatal DA are well known to reflect cue-associated expectations of reward contingencies (prediction errors; Sutton and Barto, 1981; Schultz, 2010). DA concentration within the NAc core and shell were also found to increase during acquisition of a Pavlovian cocaine-cue association (Aragona et al., 2009). Within the NAc shell, cue-evoked DA release developed during conditioning to morphine (Bassareo et al., 2007). Signaling by extracellular signal-regulated kinase (ERK) has also been shown to play a role in Pavlovian psychostimulant conditioning (Lu et al., 2006). One-trial induced Pavlovian conditioned behavioral response to cocaine has been shown to persist for at least 21 days in rats (McDougall et al., 2014). Such results lend credence to an interpretation of our results in terms of Pavlovian drug conditioning to IN-DA.

Our results have obvious relevance to the understanding of the well know placebo responses in Parkinsonian patients to medication and deep-brain stimulation. Expectation of positive outcome can enhance beneficial behavioral effects of subthalamic nucleus (STN) stimulation (Keitel et al., 2013). Placebo treatment was also found to decrease neuronal activity in the STN of Parkinsonian patients (Benedetti et al., 2004, 2016). Most relevant to our results are the findings of evidence of DA release in the striatum of PD patients to placebo using competition for [11C]raclopride binding measured by positron emission tomography (de la Fuente-Fernández et al., 2001; Lidstone et al., 2010). The decrease in striatal binding found after saline injection (placebo) presumably reflects increased DA binding to D2 receptors. Our results support these findings in the rat and provide evidence for placebo action on the DAT. It has been proposed that the release of DA may underlie placebo effects in general (de la Fuente-Fernández and Stoessl, 2002).

### Behavioral Effects

The behavioral findings below are consistent with the hypothesis of a conditioned central increase in release of DA. IN-DA treatment yielded significant differences in certain behaviors in comparison with VEH treatment only when VEH preceded IN-DA. The effects were particularly prominent in four behaviors that were decreased by IN-DA treatment, namely traveled distance, velocity, frequency of entries into the center zone and frequency of rearing.

In the animals treated with IN-DA before VEH, these behaviors did not decrease in comparison with VEH, suggesting DA conditioned behavioral effects as a likely cause, i.e., the normal behavioral response to VEH could have been masked or modified as a result of Pavlovian conditioning of the unconditioned response to the preceding IN-DA. Such an interpretation is consistent with a large literature describing Pavlovian conditioned behavioral responses and sensitization to psychoactive drugs, such as cocaine and amphetamine (Carey et al., 2014), and particularly with the results of McDougall et al. (2014), who found one-trial induced cocaine conditioned activity, which persisted for at least 21 days in rats. This interpretation is also consistent with the evidence above for a Pavlovian conditioned DA response to VEH when VEH was preceded by IN-DA.

The reduction of motor and exploratory parameters after IN-DA treatment is comparable with findings on rats obtained after L-DOPA challenge (5 and 10 mg/kg i.p.) in the same apparatus (Nikolaus et al., 2014). The findings are also consistent with reports of reduced behavioral activity after administration of this DA precursor in moderate doses (McDevitt and Setler, 1981). Our finding of reduced behavioral activity after IN-DA, seems to contradict previous findings of increased locomotor activity after the same IN-DA dose (de Souza Silva et al., 2008). The discrepancy may be accounted for by differences in procedure: in the latter study (de Souza Silva et al., 2008), rats had been pre-exposed to the open field for 30 min prior to the IN-DA or VEH trials, whereas in the present study animals were not subjected to a habituation trial prior to intranasal challenge. Accordingly, Buddenberg et al. (2008) found that IN-DA stimulated locomotor activity in a familiar, but not novel, open field. The administration of IN-DA during the prepuberal period in endogenously hyperactive rats also led to reduced motor activity in the adult (Ruocco et al., 2014).

Striatal DAT binding was significantly reduced after IN-DA relative to the VEH condition, reflecting the expected elevation of synaptic DA activity after its nasal application, as also shown by *in vivo* microdialysis (de Souza Silva et al., 2008). Attenuated behavioral response to repeated exposure to the same environment can be a consequence of behavioral habituation. Thus, the attenuated behaviors found when IN-DA followed VEH, could also reflect habituation. Since such habituation depends on a memory for an encountered environment, it is possible that IN-DA facilitated recognition of the open field, leading to enhanced habituation and attenuated behavioral activation. A possible memory-enhancing effect of IN-DA is in line with previous findings of facilitated object-place memory in rats by IN-DA application (Trossbach et al., 2014). DA is well known to play a major role in associative learning (for review, see Beninger, 1983), and specifically in working memory in humans (Luciana and Collins, 1997) and rats (Chao et al., 2013). Likely rewarding properties of IN-DA could be related to its behavioral action. Affective properties could also potentially interact with aversive or other effects of any control procedures (the carrier, handling, etc) and result in differences in behaviors observed after IN-DA vs. VEH.

Also in animals treated with VEH before IN-DA, the IN-DA treatment was followed by an increase in duration of sitting and duration of head-shoulder motility as compared to the VEH condition. These effects can be considered in relation to the reduction in motor and exploratory behaviors described above.

A significant inverse relationship was found between the neostriatal DAT binding and the duration of head-shoulder motility after IN-DA treatment and duration of head-shoulder motility and rearing after VEH treatment. For all three behaviors, lower values of exogenous ligand binding to the DAT (higher DA action) were associated with elevated expression of the particular behavior. Presumably, more availability of DA (leading to a higher reduction of DAT binding) after IN-DA, predicts a longer duration of head-shoulder motility, whereas a higher level of DA after VEH predicts a longer duration of head-shoulder motility and rearing. Reduced horizontal activity along with an increase in duration of rearing has been reported before Ruocco et al. (2014). Rearing behavior is related to exploration, attention to novelty and arousal level (Sadile, 1996). Rearing behavior has been shown to correlate with amount of *in vivo* release of DA (Biesdorf et al., 2015). Our results support such a role of dopaminergic neurotransmission in the expression of rearing, based on an inverse relationship between [^123^I] FP-CIT binding and DA binding.

## Conclusion

The results: (a) demonstrate a central action of IN-DA on the DAT in the dorsal striatum and thereby extend our understanding of the central mechanisms by which nasal DA application can influence behavior. An action of IN-DA on the availability of DA in the dorsal striatum is of high importance in light of the potential application of IN-DA as an antiparkinsonian agent (Pum et al., 2009). The action of IN-DA on DAT binding in the dorsal striatum is congruent with the IN-DA-induced DA release found previously in this site (de Souza Silva et al., 2008); (b) The results also provide first evidence of a Pavlovian conditioned DA response at the level of the DAT. Given that Pavlovian incentive conditioning is considered to be a mechanism responsible for the development and maintenance of addictive behaviors (Bassareo et al., 2007; Carey et al., 2014; Everitt, 2014), the evidence that such conditioning influences DAT binding suggests the DAT as a potential pharmacological target to influence this process. Our result also supports findings of Pavlovian conditioned DA release to placebo treatment in Parkinsonian patients and provide an animal model for further mechanistic study of such processes.

## Author Contributions

Experimental design: SN, JPH, MAdSS and HWM. Performance of imaging and behavioral studies: SN, CD. Evaluation and statistical analysis of imaging and behavioral studies: SN, CD and MB. Interpretation of findings: SN, JPH, MAdSS and HWM. Writing and editing of the manuscript: SN, JPH, MAdSS, HWM, AGS, and CM.

## Conflict of Interest Statement

None of the authors declare a conflict of interest, except for CM, who is employed by M et P Pharma AG, Emmetten, Switzerland.
